# Post‐treatment life‐trajectories among people who inject drugs who completed hepatitis C treatment with direct acting antivirals: A thematic analysis

**DOI:** 10.1111/bjhp.70005

**Published:** 2025-07-11

**Authors:** Gabriele Vojt, Lawrie Elliott, Hannah Family, Christian Sharkey, Brian Stephens, Sharon Hutchinson, Matt Hickman, John F. Dillon, Magdalena Harris, Paul Flowers

**Affiliations:** ^1^ NIHR Health Protection Research Unit in Behavioural Science and Evaluation University of Bristol Bristol UK; ^2^ National Institute for Health and Care Research, Applied Research Collaboration West (NIHR ARC West) University Hospitals Bristol and Weston NHS Foundation Trust Bristol UK; ^3^ School of Health & Life Sciences Glasgow Caledonian University Glasgow UK; ^4^ Ninewells Hospital and Medical School NHS Tayside Dundee UK; ^5^ Public Health Scotland Edinburgh UK; ^6^ Population Health Sciences, Bristol Medical School University of Bristol Bristol UK; ^7^ School of Medicine, Ninewells Hospital and Medical School University of Dundee Dundee UK; ^8^ London School of Hygiene & Tropical Medicine London UK; ^9^ School of Psychological Sciences & Health University of Strathclyde Glasgow UK

**Keywords:** direct acting antiviral, hepatitis C, long term outcomes, people who inject drugs, qualitative, substance use

## Abstract

**Background:**

Hepatitis C Virus is a global health burden, particularly affecting people who inject drugs. Direct‐acting antiviral treatment can cure almost all people infected with hepatitis C. However, the longer‐term impacts on within people's life trajectories are unknown, although some suggest it may lead to fundamental behavioural changes.

**Design:**

A cross‐sectional exploratory retrospective qualitative design.

**Methods:**

We interviewed 30 people who inject(ed) drugs and who had previously been treated with direct‐acting antivirals on average 18 months before the interview (range: 12–24 months). Most were male, aged 36–55 years, and had been treated in urban community‐based needle and syringe programmes in Scotland. All interviews were semi‐structured, conducted over the phone by either peer or academic researchers, and analysed thematically.

**Results:**

We present three discernible post‐treatment life‐trajectories. Firstly, ‘*A cure that was like totally new life’* involved identity transformation, often associated with controlled or zero drug use, and improved physical and mental health. Secondly, ‘*There isn't much afterwards*’ comprised poor quality of life and social isolation, often due to participants' disengagement from former drug use and support networks. Thirdly, ‘*I'm getting tested every year now’* detailed a few life changes but normalized hepatitis C testing and treatment.

**Conclusion:**

Our thematic analysis reinforces the positive impacts on mental well‐being, physical health and self‐transformation of direct‐acting antiviral treatment in the short‐ and long‐term. However, there is an urgent need for psychosocial interventions to maintain health behaviour changes and self‐management, and social support for individuals post hepatitis C treatment.

**Statement of Contribution**

**What is already known on this subject?**

- Direct‐acting antivirals (DAAs) enable a quick and easy cure of hepatitis C virus (HCV) among people who inject(ed) drugs (PWID). This may lead to wider behavioural health changes such as engagement with drug treatment and focused harm reduction.
- Some evidence suggests that short‐term outcomes post HCV DAA treatment are improved mental and physical health, personal transformation and behavioural changes such as reduced or abstinent drug use. However, few studies have examined the long‐term impacts of HCV DAA treatment (>18 months post‐treatment completion).
- There is a need to better understand the emotional and social responses to HCV DAA treatment to enable effective intervention development, with the aim of maintaining achieved behavioural changes in the long term.

**What does this study add?**

- The thematic analysis identified three life trajectories among a group of 30 PWID, comprising people who are actively injecting drugs and those who are abstinent from drugs, and who completed HCV DAA treatment up to 24 months ago.
- Findings highlight diverse impacts in the short‐, mid‐ and long‐term, ranging from positive identity transformation, improved well‐being and self‐efficacy to poor mental health and social isolation.
- The design and development of focused psychological and social interventions to support PWID alongside and following HCV DAA treatment are essential to HCV elimination efforts.

## INTRODUCTION

Hepatitis C virus (HCV) is a significant global health burden that can be effectively treated with direct‐acting antiviral (DAA) medication. DAAs have a high HCV cure rate (>95%) over a brief treatment course (8–12 weeks) and with little to no side effects (Pawlotsky et al., 2020). HCV is a blood‐borne virus, with the use of unsterile injecting equipment as a primary transmission route in many countries. People who inject drugs (PWID) are, therefore, a key population for enhanced DAA access (Shiffman, 2018). The aim is to eliminate HCV by 2030 (World Health Organization, 2016) with DAA treatments believed to be instrumental in achieving this (Hellard et al., 2020). DAA treatment is highly cost‐effective (Verma et al., 2021) and can reduce HCV prevalence when scaled up in low‐threshold services (Fraser et al., 2018; Palmateer et al., 2022). In terms of HCV reinfection, some studies report an increase in reinfection rates post DAA treatment (Carson et al., 2022; Yeung et al., 2022), yet others suggest that the scale up of HCV testing and treatment can lead to low population HCV reinfection in the long term (Hajarizadeh et al., 2020), in particular when coupled with accessible harm reduction such as opioid agonist treatment (OAT) and needle and syringe provision (Martinello & Matthews, 2024). Harm reduction includes ‘practical strategies and ideas aimed at reducing negative consequences associated with drug use’ (National Harm Reduction Coalition, 2020). In relation to emotional and behavioural responses, completing DAA treatment can lead to positive personal transformation (Harris & Rhodes, 2018), may facilitate harm reduction (Caven et al., 2019), alleviate stigma and support HCV disclosure (Krzeczkowska et al., 2021), increase self‐management (Bailey Jr. et al., 2021) and improve health‐related quality of life (Elbadry et al., 2024).

Key psychological health theories suggest that critical life events are likely catalysts for wider behavioural changes (Becker, 1974; Bury, 1982; Leventhal et al., 2016; Prochaska & Velicer, 1997). Existing studies on behaviour change following HCV DAA treatment are predominantly based on qualitative research designs, and are located in Australia, the UK, Switzerland and Canada (e.g., Goodyear et al., 2021; Goutzamanis et al., 2021, 2022; Guggisberg et al., 2022; Harris, 2017; Madden et al., 2018; Pourmarzi et al., 2020; Richmond et al., 2018). Almost all studies report participants' *expected* improvement in mental and physical health (Goodyear et al., 2021; Madden et al., 2018), reconnection to others (Harris, 2017; Madden et al., 2018), increased self‐efficacy in health management (Madden et al., 2018; Pourmarzi et al., 2020) and either a return to normality (Harris, 2017) or the sense of a new start in life (Madden et al., 2018). A few studies focus on post‐HCV DAA treatment *experiences*, ranging between immediately (Goutzamanis et al., 2021; Guggisberg et al., 2022) and up to 18 months after treatment completion (Harris, 2017; Madden et al., 2018). Here, most participants report better quality of life (Goutzamanis et al., 2022; Harris, 2017), and improved well‐being and physical health (Goodyear et al., 2021; Goutzamanis et al., 2021; Guggisberg et al., 2022; Richmond et al., 2018). Harris (2017) and Goodyear et al. (2021) discuss the gap between participants' expectations, hopes, and experiences post‐HCV DAA treatment. Both highlight the ongoing experience of stigmatization and wider systemic barriers to social integration despite being HCV cured, e.g., in terms of housing and employability. Gaining insights into long‐term responses to DAA treatments may therefore help in developing a better theoretical understanding of the barriers and facilitators to behaviour change. This, in turn, enables the development of effective interventions to maintain behaviour change (Kwasnicka et al., 2016), self‐management, detection and engagement with treatments, facilitating individual health (e.g., Araújo‐Soares et al., 2019). However, research follow‐up times post‐HCV treatment are often short (<12 months) (Goodyear et al., 2021; Goutzamanis et al., 2021; Madden et al., 2018; Richmond et al., 2018). Reported post‐HCV DAA treatment experiences may be limited in diversity, depth and transferability of findings. For example, outcomes are not necessarily HCV DAA treatment‐specific, but rather mixed with participant experiences from interferon medications (Harris, 2017), or the study conclusions on treatment impact may not apply, as the majority of participants had not completed HCV DAA treatment yet (Madden et al., 2018). Some studies fail to identify the status of participants (e.g., currently vs. historically injecting) (Richmond et al., 2018), and some findings are based on people described as ‘stabilized’ on OAT, thus reflecting those with potentially better outcomes (e.g., Pourmarzi et al., 2020). Long‐term experiences are often presented for the full sample and tend to solely highlight commonalities rather than differences across the sample, although there are some exceptions to this general trend (i.e., Goodyear et al., 2021; Goutzamanis et al., 2022; Harris, 2017).

In this paper, we use the concept of life trajectory to capture the experiences of longer‐term changes and social processes (e.g., Riemann & Schütze, 1991) among people who completed HCV DAA treatment. We address a single research question: How are the longer‐term life trajectories of those who complete HCV DAA treatment understood?

## METHODS

### Design

We conducted a cross‐sectional retrospective qualitative study with PWID who completed HCV DAA treatment in a range of low‐threshold community HCV services (pharmacies, needle and syringe programmes, drug treatment services) and prison in Tayside, Scotland.

### Participants

We interviewed a cross‐section of 30 people who had *ever* injected drugs, including people who currently injected at the time of the interview, and those who had ceased injecting (potentially temporarily) as they were either on OAT or in prison, and who had completed HCV DAA treatment in the community or in prison. The mean and median times between HCV DAA treatment completion and interview were 18 months (SD = 3.03 months), ranging from 12 to 24 months. Table 1 provides descriptive data on the sample interviewed.

**TABLE 1:** Descriptive data on the sample's HCV treatment course.

	Number (%)
Self‐reported gender	
Female	6 (20.0%)
Male	24 (80.0%)
Age	
26–35 years	6 (20.0%)
36–45 years	15 (50.0%)
46–55 years	8 (26.7%)
Missing	1 (3.3%)
Residence/Location	
Urban	25 (83.3%)
Rural	5 (16.7%)
Self‐reported injecting of drugs at time of interview	
Yes	17 (56.7%)
Healthcare setting where HCV treatment took place	
Prison	2 (6.7%)
Pharmacy	4 (13.3%)
Drug Treatment	5 (16.7%)
Needle and Syringe Programme	19 (63.3%)

### Procedure

#### Recruitment

Eligible HCV treatment completers were identified and recruited via HCV nurse specialists using a set of inclusion criteria: the person must have ever injected drugs (including currently, i.e., at time of recruitment, or was on OAT); was aged 18+ years; and had completed HCV treatment via DAAs at least 12 months ago in prison or in community‐based HCV services (needle and syringe programmes, pharmacy or drug treatment) in Tayside, Scotland. The focus, therefore was on treatment completion rather than being cured. HCV nurse specialists provided verbal information on the study and handed information sheets to potential participants in a range of outreach settings (needle and syringe programmes, drug treatment, pharmacy). Those who expressed an interest in taking part in an interview gave permission for the HCV nurse specialist to share their contact details with the researchers and to provide demographic data in terms of HCV treatment settings, gender, age category, injecting status at the time of interview and residence in urban or rural areas. Researchers then contacted service users to arrange and conduct the interview. All interviewees received £20 in vouchers as a token of appreciation for their time and help.

#### Topic guides and data collection

We designed a topic guide (see supplementary file) that focuses on the interviewee's experience of completing HCV DAA treatment, the support received and the subsequent quality of life in terms of health, substance use, social inclusion and overall well‐being. We sense‐checked the topic guide with peer researchers, i.e., people with lived experience of substance use and HCV, at the Scottish Drugs Forum (SDF). All data collection was conducted through individual telephone interviews (necessarily due to Covid restrictions) either led by local, qualitatively trained peer researchers (SDF) or a qualitative academic researcher between August 2020 and March 2021. Interviews conducted by peer researchers (*n* = 7, 23.3%) were in a 2 2‐1 format with a member of SDF staff co‐facilitating where required. Most interviews (*n* = 23, 76.7%) were conducted by one of two experienced academic qualitative researchers (GV, LE) because they were able to respond more flexibly and faster to often impromptu interviews with recruited service users. Interviews lasted a mean of 28 min (SD = 10 min), with a range of 11 to 48 min. We ceased collecting data when we felt we had reached data saturation and encountered no new narratives. We also ensured that we had met the sample size stated in the protocol (Hickman et al., 2019) with regard to available resources, ethical approval and funders' requirements. Interviewees were asked to verbally give informed consent as part of the audio‐recorded interview. Throughout all interviews, we also sought continuous consent and ensured interviewees were aware of their right not to answer questions. On one occasion, an interviewee chose to withdraw their consent before the start of the interview because they personally knew the peer researcher and did not feel comfortable answering questions. This person is not part of the total interview sample.

### Data analysis

All interviews were pooled and analysed inductively together using thematic analysis (Braun & Clarke, 2019 ). Broadly, we followed Braun and Clarke's five‐phased approach: (1) GV read and re‐read all anonymized transcripts to familiarize themselves with the data, (2) GV open‐coded transcripts by reflecting and discussing the underlying narratives and reported experiences, (3) next, GV organized these initial open codes into larger analytic entities (i.e., themes) and then determined the characteristics, i.e., subthemes, that underpinned each theme. Lastly, (4) GV defined themes and subthemes by contrasting reported changes post‐HCV DAA treatment in the short‐ and longer‐term. All themes and findings were discussed and refined with a senior qualitative researcher (PF) and with the wider research team.

### Ethics approval

Ethical approval was obtained from the East of Scotland Research Ethics Service REC 1 (18/ES/0128) as part of a wider research programme, Evaluating the Population Impact of Hepatitis C Direct‐Acting Antiviral Treatment as Prevention for People Who Inject Drugs (EPIToPe).

## RESULTS

**FIGURE 1:**
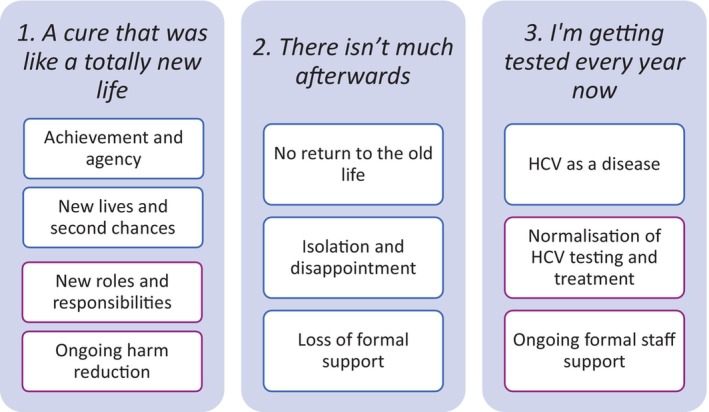
Themes relating to post‐treatment life‐trajectories.

The analysis detailed three important post‐treatment life‐trajectory themes and associated subthemes. As interviews were conducted on average 18 months post‐treatment, participants' accounts span both the immediate short‐ and longer‐term impacts of HCV DAA treatment. Figure 1 details the themes and their subthemes: (1) ‘*A cure that was like a totally new life’*, (2) ‘*There isn't much afterwards*’ and (3) ‘*I'm getting tested every year now’*.

In the following sections, illustrative quotes are accompanied by a pseudonym, reported gender and the setting where HCV DAA treatment was completed. We used abbreviations where ‘PR’ indicates prison, ‘PH’ means pharmacy, ‘DT’ refers to drug treatment and ‘NSP’ stands for needle and syringe programmes.

### 1. ‘A cure that was like a totally new life’

This theme is composed of subthemes that focus on the ways in which becoming HCV‐free as a result of HCV treatment was linked to other positive life changes and overall improvements in physical and mental health. When people discussed this positive, transformatory life trajectory, a sense of achievement and subsequent personal growth were often mentioned, as well as a wide range of short‐ and long‐term changes. In the short term, there was a clear sense of achievement, increases in personal agency, feeling ‘free’, energized, motivated and being given a fresh start. In the longer term, this trajectory was associated with changes to key relationships, roles, new responsibilities and the maintenance of harm reduction in relation to both drug use and HCV reinfection prevention.

The first subtheme ‘*achievement and agency’* focuses on the ongoing psychological mechanisms participants imagined to be associated with this positive, transformatory life trajectory. Here, completing HCV DAAs was understood as representing a significant personal achievement and the realization of an important goal. This short‐term achievement (i.e., starting and completing a time‐dependent treatment course either autonomously or with the support of community pharmacy) must be understood in the context of the lives of those who inject drugs. Therein, the complexity of multiple disadvantages, stigma and chronic or recurrent health conditions means self‐efficacy can be low (Yang et al., 2019) and a sense of achievement can be rare, especially within healthcare domains. Therefore, in this trajectory, completing treatment was associated with pride and empowerment. The subtheme also highlighted a deep sense of relief, the lifting of shame, feelings of being motivated and energized. Together, these short‐term transformations were understood as being catalytic in shaping wider life changes:

*‘I got this, the hepatitis, the first time. I've never been so scared for myself before. I thought I was going to die, so I had to see it* [HCV DAA treatment] *through, and I now know I can do it. It's actually easy. I can do anything.’* (Jack, male, NSP)

*‘For me this* [HCV treatment] *has gave me what I needed, that wee kick*. […] *And at the end I did feel a bit like wow, that's it, it's* [HCV treatment] *done now. Clean start. Feeling lucky, lucky to be alive*. (Otto, male, DT)

The second subtheme ‘*new lives and second chances’* focuses on reconnecting to older relationships, in particular with people's families (parents, siblings, but also children) and the longer‐term development of new relationships. For example, in the illustrative extract below, we see the profound sense of opportunity associated with this trajectory:

*‘It was a new life, because I knew I was clean and I didn't have it* [HCV] *and I could go and … I've been single for a few years now. And now I could go and get myself a girlfriend and don't have to worry about needing to tell her or stuff like that*. [….] *I got a cure that was like just a total new life. I knew it was just totally like a new life.’* (Greg, male, NSP)

For others, this positive life trajectory was seen as pivotal in reconnecting the self with former habits and routines in positive, life‐changing ways. For example, following HCV treatment, many participants felt liberated to re‐engage in social activities. Out of fear of onward transmission, participants had chosen to stay away from their families and partners or changed their behaviours to protect those around them.

*‘It* [HCV treatment and cure] *was completely a second chance at life again. I started going out to the snooker and that, because I stopped going. I used to go to the snooker and that a lot and the darts, and I stopped doing that for ages. Then after the treatment and I got the ‘all clear’ I started going out and socialising a lot more, going to the snooker and that a lot more. Life was just better.’* (Luke, male, NSP)

This third subtheme, ‘*new roles and responsibilities’* showed changes such as the establishment of new and rewarding roles and responsibilities. These included being a partner, a parent or grandparent, an employee in a new job, or as a (volunteer) peer worker in hepatitis C and substance use.

*‘It's* [HCV treatment] *made my life a lot easier, a lot better. Now I can go and sit with people* [as a peer worker] *and maybe they say they've got it* [HCV] *and blablablablabla, and I can say well I've not got it, you know what I mean. And I'll say, well I've done the* [HCV DAA] *treatment, know what I mean, I was able to sit and tell people look, it's easy, you know what I mean, to explain to them how I done it.’* (Susan, female, NSP)

In the final subtheme of this life trajectory, ‘*ongoing harm reduction’*, participants talked about how this sense of achievement, pride, agency and change also impacted drug use and harm reduction. The experience of effective treatment was understood to be catalytic in shaping practices such as ongoing re‐testing and not sharing injecting equipment.

*‘That's actually come out from having hep C a number of times. Because I used to leave equipment with other people…and it* [positive HCV diagnosis] *just made me not trust other people, because obviously they've been using my equipment. I always carry my own equipment with me, every single time since*. […] *I don't hand equipment onto nobody, anymore, full stop. I keep my own stuff.’* (Harris, male, NSP)

This theme, *‘A cure that was like a totally new life’* depicts a life trajectory focused on the positive transformative potential of completing HCV DAAs. It has detailed, wide‐ranging short‐ and long‐term effects. It also offers insights into a range of mechanisms, both psychosocial and structural.

### 2. ‘There isn't much afterwards’

In contrast to the theme above, here we focus on our analysis of participants' talk relating to largely negative post‐treatment life‐trajectories. We present three subthemes that chart a trajectory that eventually leads to a decline in people's mental well‐being and quality of life. While people talked of this trajectory in ways that highlighted both personal and wider social change in the short term, these changes did not lead to a blossoming of new possibilities but to a longer‐term contraction of people's social worlds.

The first subtheme, ‘*no return to the old life’* highlights how, after having attained a sense of achievement at being HCV free, attention and personal agency subsequently focussed on remaining HCV‐ and, for some, drug free. In this trajectory, as in the one above, the short‐term immediate effects of treatment included initial feelings of relief and feeling energized. However, while striving to remain HCV‐free, this trajectory highlights how in the medium‐ and longer‐term, people may focus on distancing themselves from their old lives rather than building new lives. The illustrative extract below shows this sense of agency, change and rejection of previous friends and acquaintances:

*‘I've got friends I can go to if I want, but I choose not to ‘cause they're all taking drugs. So that's…mate, that makes it harder. It's not that I can't sit with them when they take drugs, I can, but I'm not going to sit round idiots that are taking drugs when I've done that and stopped it. So why should I sit about people that still do it*. […] *I'm trying to do better than them. But trying to do better than them, you've got to change. It's not just changing what you do from day‐to‐day, it's changing your pals, it's changing your lifestyle, it's changing everything. It's not just one thing.’* (Finn, male, NSP)

The second subtheme ‘*isolation and disappointment’* highlights how, post‐treatment, the avoidance of previous social networks can lead to profound social isolation. Finn, in particular, emphasizes the disconnect from previous networks and, by extension, his previous self, while Millie voices her experience of isolation and loneliness following DAA treatment.

*‘Now I've trapped myself in the house basically, ‘cause everybody…and sorry for saying, everybody I know is a fucking junkie. And I don't like that word* […] *I hate that word. But that's what they are. And that's what I was.’* (Finn, male, NSP)

*‘I'm happy that I've not got, like, hep C anymore. But there isn't much afterwards, I don't know. Because I just sit in my house on my own and just watch a bit of TV and, like…I like doing Sudokus. So I do them. And I just watch a bit of TV and just sit in my house all day.’* (Millie, female, PH)

While personal agency is associated with this trajectory, it is also important to acknowledge the role of more geographical, social and structural factors. Within local communities, people were often stigmatized as known drug users. These social dynamics were seen as important because *‘junkie’* reputations curtailed the possibility of new relationships developing or new roles and responsibilities being acquired (such as employment). Reputation for past drug use also presented new vulnerabilities for people trying to remain HCV‐free by avoiding drugs, as in their neighbourhoods, dealers could target them. Together, these factors were understood to lead to isolation and sometimes disappointment and depression.

*‘It's* [HCV DAA treatment] *helped by getting me off* [heroin] *and it's hindered by I've changed my fucking whole life now. I've got rid of everybody. And now I'm sitting in a two bedroomed up and downstair house on my own*. […] *I've got absolutely nothing.’* (Finn, male, NSP)

The final subtheme ‘*loss of formal support’* shows how the contraction of people's social worlds in this life trajectory could also include the loss of formal sources of support and help. When people are injecting drugs, they may use a range of health care services, such as drug services or needle and syringe programmes. In these settings, they may interact with a variety of professionals (e.g., HCV nurse specialists, peer workers) and other PWID. As the previous subtheme highlighted, once HCV has been treated effectively, people may actively avoid being exposed to interacting with people and places from their former lives. This subtheme highlights how these dynamics may be associated with further loss of social support.

[So, who do you have for support?] ‘*Nobody.’* [What about people at [support hub]? Are you in touch with the staff, maybe?] *‘No, I've no been really back to the* [support hub] *since I obviously finished my* [HCV] *treatment? I came back negative, and I'm no taking heroin anymore, I telt you that, so why would I go back there? I'm trying to stay away from people who use, and with the* [support hub] *and the needle exchange, it's not good for me.’* (Diane, female, DT)

For some, reduced support was exacerbated by wider life changes. For example, Raab details a lack of support following release from prison into the community:

*‘They don't really help you much, you know what I mean, when you're outside. It's* [sharing injecting equipment] *inevitable; it's going to happen*. [How is it inevitable?] *It's…there is nothing for you. I have nothing. I am in a hostel just now. I can't sit here all day long. I can't not go and see people. What else am I supposed to do? There is no help, I don't know. Nobody tells you how to stay away from everything you know.’* (Raab, male, PR)

This theme *‘There isn't much afterwards’* shows a life trajectory detailing the way initial positive changes after treatment can be followed by longer‐term negative changes. The theme offers a way of understanding how personal agency, social and geographic circumstances can conspire to constrain rather than release longer‐term potential in people treated with DAAs.

### 3. ‘I'm getting tested every year now’

The final life trajectory theme focussed on subthemes outlining limited personal or social changes related to HCV DAA treatment. It highlights a continuation of the status quo in terms of well‐being and quality of life. However, together the subthemes depict a positive sense of HCV as a medical and treatable infection, with repeat testing and treatment having been normalized, and participants being able to access healthcare staff for continuous support.

The first subtheme *‘HCV as a disease’* is underpinned by people's understanding of HCV as a treatable infection. The short‐term impacts of HCV treatment were discussed in terms of improved physical health and feeling relieved. Longer‐term treatment‐related changes were not anticipated, and major life changes were not attributed to HCV treatment but to wider life changes (e.g., ageing) if mentioned at all:

*‘I just felt a lot better in myself* [after HCV DAA treatment]. *I hope to stay more healthier, more healthier*. [How do you mean healthier?] *I wasn't feeling so good before it, and then after it, great, you know?* [And did you change anything else?] *No, not really, no. I still just stayed the same. Yeah, I just stayed doing what I always do.’* (Adam, male, DT)

*‘It doesn't matter how many times you get it* [HCV], *they told me, so it doesn't really interest me. You know you can get rid of it and that's half the battle*. [Are there any long‐term benefits of having done the Hep C treatment for you?] *No. Definitely not, definitely not. Why should there be anything? I don't think it matters.’* (Eli, male, DT)

The second subtheme *‘Normalization of HCV testing and treatment’* highlights how testing and treatment can become habitual and routine. In this subtheme, participants did not connect testing or treatment to a sense of personal agency or achievement:

*‘It's like, you know, I'll admit, I do still inject drugs, now, but not to the extent that I used to, nowhere near* […] *I go in for testing. I get it done once a year, every July. It's just something that I do, and I know, when I first got tested for hep C, or when I first found out I was hep C, I remember, it was in July. And so, once every year now, in July, I just get my blood test done, just to make sure I'm clean and clear.’* (Harris, male, NSP)

*‘I've done it* [HCV DAA treatment] *three times*. [What was that like?] *It's easy. It's easy to get tested, it's easy to get treated, it's easy as anything.’* (Ian, male, NSP)

The third subtheme *‘Ongoing formal staff support’* was also linked to the overall normalization of HCV testing and treatment. Here, ongoing connections and support from staff, such as HCV nurse specialists, were key in enabling people to engage in regular testing and repeat treatment when participants reported having been at risk. Non‐judgmental attitudes were often mentioned as being particularly important in the normalization of testing and treatment, and contributed positively to people's enablement to access healthcare:

*‘Yeah, I get my bloods checked every four months* [post HCV treatment]. [And what's that like for you?] *‘I get a lot of support from* [HCV nurse specialist], *so I don't mind, I think it's important to get checked, and I know I can trust him.’* (Oliver, male, NSP)

*‘Oh they're all lovely where we get checks for the Hepatitis C in* [city]. *Staff are amazing, non‐judgmental. You know, because I have it in my head like ‘oh I've fucked it* [HCV treatment] *up again, I've not completed it again, it's a lot of money and stuff’ but all the staff are like lovely in there eh*. […] *There isn't any like oh, you should have done this, and you shouldn't have done that, do you know what I mean? They're just happy to see you and to get tested again and get back on the treatment if you require it, do you know what I mean?’* (Julia, female, NSP)

This life trajectory theme *‘I'm getting tested every year now’* was characterized by a lack of changes attributed to HCV treatment. Participants' talk highlighted the routine nature of testing and treatment and a clear appreciation that reinfection and a new treatment cycle were possible. In contrast to the previous two life trajectory themes, there was no sense of achievement and pride following HCV treatment. Instead, the current trajectory was shaped by a normalization of HCV testing and treatment. Formal staff support was seen as enabling and ensuring that reinfections were addressed without feelings of shame or guilt.

## DISCUSSION

In this paper, we identified three HCV treatment trajectories in a qualitative study with PWID who were treated with DAAs in the context of a coordinated scale‐up of HCV treatment across a range of community outreach settings. These trajectories were associated with a range of impacts and subsequent transformative changes, both psychosocially and structurally. People's treatment journeys had been diverse and heterogeneous, yet for most, short‐term impacts typically described a sense of pride and feeling good with improved immediate physical and mental health. In the life trajectory ‘A cure that was like a totally new life’, longer‐term impacts comprised fundamental and positive life changes; with the self‐managed completion of their HCV DAA treatment, participants talked of feeling enabled and empowered to commit to further changes. The findings from our second trajectory, ‘there isn't much afterwards’ mirror these experiences up to the short‐ and medium‐term. In the longer term, however, the lack of formal support and a disconnect from known networks led to social isolation and poor mental health. Lastly, the trajectory ‘I'm getting tested every year now’ outlines limited perceptions of life changes attributed to HCV DAA treatment per se, yet, participants' narratives reflect immediate, short‐term benefits (better physical health) from having been cured of HCV. The longer‐term impacts of HCV testing and treatment highlighted the normalization and the benefits of ongoing formal staff support in harm reduction.

Our findings reiterate insights from existing key literature in terms of theory, i.e., the common‐sense model of self‐regulation (Hagger & Orbell, 2021; Leventhal et al., 2016) and biographical disruption theory (Bury, 1982), and HCV treatment outcomes and experiences including those reported from previous HCV treatments (i.e., interferon based). In particular, our trajectory patterns 1 and 2 (‘A cure that was like a totally new life’ and ‘There isn't much afterwards’) reflect some of the positive changes following HCV DAA treatment reported by Harris (2017) and Goutzamanis et al. (2021). Themes around second chances and starting a new life meant the transformation of the self through new roles and responsibilities, creating new and reconnecting to old relationships, routines and habits. People's sense of achievement and pride highlight key opportunities and could inform the development of intervention contents and mechanisms for healthcare professionals, key workers and significant others to use effectively. This sense of achievement post HCV DAA treatment may be surprising given the ease, speed and the reported lack of side effects in DAA treatment when compared to the considerably longer (24–48 weeks) and more difficult (multiple side effects) interferon‐based therapies. Here, participants reported achievement and having ‘earned’ their cure (Artenie et al., 2017; Batchelder et al., 2017). Our findings make sense when considering previous literature and theory such as Bury's theory of chronic illness (Bury, 1982) and ‘illness identity’ (Van Bulck et al., 2019), i.e., the ways in which participants discussed the influence of HCV on their sense of self, their person and social relationships. The feared and, for some, actual chronicity of (untreated) HCV overshadows any ease and speed with which HCV DAA treatment may be experienced, and hence the impact of having fully engaged and completed a DAA treatment course is immense on some people's selves (Bury, 1982; Whiteley et al., 2018).

The way some participants talked about being able to start a new life – free of HCV – aligned with the survivor and ‘recovery’ literature where identity transformations are reported following major life events (e.g., surviving trauma, life‐threatening illnesses, substance use disorder) (Pytell et al., 2022). People's narratives on hope, connectivity and new beginnings echoed principles of the 12‐step fellowship, which is a therapeutic programme aimed at recovering from substance use via personal transformation (Dekkers et al., 2020). Across our life trajectories, HCV DAA treatment experiences were often entangled with how people viewed and managed their social identities within the context of drug use (Dingle et al., 2015). That is, the impacts of HCV DAA treatment and the impacts of reduced/ controlled/ abstinent drug use were described in interchangeable ways. Related to this, we found that many participants believed that effective HCV treatment required (temporary) reduction or abstinence from drug use, which Vega et al. (2021) reported as well. This is concerning because it is inaccurate, increases mortality, hinders healthcare access and negatively impacts wider public health (Grebely et al., 2018). Further, we found HCV DAA treatment was associated with participants' implementation of harm reduction strategies such as not sharing injecting equipment, mirroring conclusions by Goodyear et al. (2021) and Goutzamanis et al. (2021). The positive uptake of keeping safe was also evident in trajectory 3 (‘I'm getting tested every year now’), with individuals engaging in repeat and normalized HCV testing and treatment, i.e., in the absence of service‐level stigma. Social connectivity to healthcare staff who advocate a non‐judgmental approach is key here. This adds to conclusions from Hajarizadeh et al. (2020) underlining that continuous and repeated harm reduction advice, reminders and conversations—regardless of drug status and treatment engagement—may be fruitful to avoid HCV reinfection, increased financial and personal costs such as fear of (repeated) HCV stigmatization and negative health impacts.

Importantly, our findings can help to contextualize wider research findings via the three life trajectory themes. For example, McDonald et al. (2023) conducted a longitudinal quantitative study on quality of life in the same conurbation in Scotland. McDonald and colleagues documented an initial patient‐reported improvement in quality of life among 83 people who inject(ed) drugs immediately following HCV treatment. However, improvements were not sustained at 12 months follow‐up. This makes sense when considering our ‘there isn't much afterwards’ and ‘I'm getting tested every year now’ trajectories. Similarly, our trajectories reinforce the conclusions from Hamill et al.'s (2023) large population cohort quantitative study on a sample of 21,790 HCV treatment completers from British Columbia, Scotland and England. The authors report that the overall mortality is higher when compared to the general population (OR = 2.96), i.e., despite HCV treatment being a possible catalyst for personal transformation and life changes. These results map onto our findings, in particular in respect to the need for extended and long‐term public health support, regardless of drug using status.

## Implications for policy, services and research

In the short term, findings from the trajectory ‘I'm getting tested every year now’ underline the positive impact of normalising HCV on people's health behaviours. Public health messaging and awareness raising of regular testing should be designed to support normalization and highlight the ease and speed with which testing and treatment can be conducted. Existing harm reduction interventions and advice should reiterate the effectiveness of HCV treatment regardless of drug using status. This should be highlighted among PWID and healthcare professionals, in particular doctors, drug treatment workers, HCV nurse specialists and pharmacists, with the aim of educating and supporting people at risk of HCV. In the mid‐term, existing third sector organizations providing formal support, referrals to mental health and drug treatment services, harm reduction advice and education should receive sufficient funding to extend support provision by offering PWIDs a wider range of voluntary and employed roles and responsibilities. In addition, focused psychological and social interventions should be designed to support PWID wishing to reduce their drug use, improve and maintain well‐being and redefine the self. In the long term, current recovery‐focused groups such as fellowships and third sector organizations facilitating recovery by providing voluntary roles such as peer workers address some of our findings. However, findings from the trajectory ‘there isn't much afterwards’ highlight the need for long‐term interventions supporting mental health, meaningful employment, training and education opportunities to maintain achieved behaviour changes, agency, confidence and self‐efficacy (Black, 2021). Ongoing staff support and care, either in the form of a trusted keyworker or a peer recovery coach (Eddie et al., 2019), to facilitate integration into new networks and communities, are also key. Further research is therefore crucial: longitudinal quantitative studies are required to validate the identified life trajectories and possible outcomes. Motivational interviewing, support and decision‐making aids should be designed for healthcare professionals to identify people's readiness to implement and maintain behavioural changes at different stages. Intervention development studies, focusing on psychological and social support, are needed, with the possible extension of trials and cost‐effectiveness evaluations of these interventions in the long term.

## Strengths and Limitations

Strengths include the involvement of peer researchers, the third sector and input from a multidisciplinary team (epidemiology, health psychology, behaviour change analyst, hepatitis C professionals). Our sample was diverse and reflected the richly nuanced and contextual experiences of people currently injecting drugs as well as those considering themselves recovered, which ranged from not injecting drugs to being abstinent. Interviewees had been treated for HCV at different durations in the community, with up to 24 months post‐HCV treatment. Our limitations centre around recruiting primarily from a needle and syringe programme where most of our participants had been tested and treated. This is likely to have shaped our findings. Further, our findings are a cross‐sectional snapshot of people's experiences, and therefore, the identified life trajectories require sense‐ and member‐checking in the long term.

## CONCLUSION

This study strengthens the evidence that HCV treatment via DAAs can act as a powerful catalyst for various psychological and structural changes in the short‐ and long‐term. In line with the biopsychosocial model, our findings highlight the need to address psychological and social challenges when treating hepatitis C. To maintain longer‐term behavioural health changes, interventions should build on people's sense of achievement, agency and self‐efficacy following HCV treatment, but also support those who experience social isolation and poor mental health following disengagement from their former drug‐using networks. At large, our findings imply that the aim of eliminating HCV may be at stake unless better psychological and social interventions are provided.

## AUTHOR CONTRIBUTIONS

**Gabriele Vojt:** Writing – original draft; investigation; writing – review and editing; project administration; formal analysis; data curation; visualization. **Lawrie Elliott:** Conceptualization; investigation; writing – review and editing; supervision; methodology; validation; funding acquisition. **Hannah Family:** Writing – review and editing; resources. **Christian Sharkey:** Resources; writing – review and editing. **Brian Stephens:** Resources; writing – review and editing. **Sharon Hutchinson:** Funding acquisition; writing – review and editing; conceptualization. **Matt Hickman:** Funding acquisition; writing – review and editing; conceptualization. **John F. Dillon:** Writing – review and editing; funding acquisition. **Magdalena Harris:** Writing – review and editing; conceptualization; funding acquisition. **Paul Flowers:** Conceptualization; methodology; writing – review and editing; writing – original draft; supervision; validation; funding acquisition.

## CONFLICT OF INTEREST STATEMENT

The authors have no conflict of interest to declare.

## Supporting information


Data S1.

## Data Availability

Research data are not shared.

## References

[bjhp70005-bib-0001] Araújo‐Soares, V. , Hankonen, N. , Presseau, J. , Rodrigues, A. , & Sniehotta, F. F. (2019). Developing behavior change interventions for self‐management in chronic illness: An integrative overview. European Psychologist, 24(1), 7–25. 31496632 10.1027/1016-9040/a000330 PMC6727632

[bjhp70005-bib-1001] Artenie, A. A. , Zang, G. , Daniel, M. , Fortier, E. , Jutras‐Aswad, D. , Puzhko, S. , & Bruneau, J. (2017). Short‐term injection drug use changes following hepatitis C virus (HCV) assessment and treatment among persons who inject drugs with acute HCV infection. The International Journal on Drug Policy, 47, 239–243. 28587944 10.1016/j.drugpo.2017.05.033

[bjhp70005-bib-0002] Bailey, D. E., Jr. , Muir, A. J. , Cary, M. P., Jr. , Ammarell, N. , Seaver, S. , Scirica, E. , Mah'moud, M. , & Anderson, R. A. (2021). Adaptive challenges and family support: Patient self‐management during treatment for chronic hepatitis C. Nursing Science Quarterly, 34(4), 405–412. 34538181 10.1177/08943184211031602

[bjhp70005-bib-1002] Batchelder, A. , Cockerham‐Colas, L. , Peyser, D. , Reynoso, S. , Soloway, I. , & Litwin, A. (2017). Perceived benefits of the hepatitis C peer educators: A qualitative investigation. Harm Reduction Journal, 14, 1–7. 28962652 10.1186/s12954-017-0192-8 PMC5622540

[bjhp70005-bib-0003] Becker, M. H. (1974). The health belief model and personal health behavior. Health Education Monographs, 2, 324–508.

[bjhp70005-bib-0004] Black, C. (2021). Review of drugs part two: prevention, treatment and recovery. *Independent Report*. https://www.gov.uk/government/publications/review‐of‐drugs‐phase‐two‐report/review‐of‐drugs‐part‐two‐prevention‐treatment‐and‐recovery#foreword (accessed 5 September 2022).

[bjhp70005-bib-0051] Braun, V. , & Clarke, V. (2019). Reflecting on reflexive thematic analysis. Qualitative Research in Sport, Exercise and Health, 11(4), 589–597.

[bjhp70005-bib-0006] Bury, M. (1982). Chronic illness as biographical disruption. Sociology of Health & Illness, 4(2), 167–182. 10260456 10.1111/1467-9566.ep11339939

[bjhp70005-bib-0007] Carson, J. M. , Dore, G. J. , Lloyd, A. R. , Grebely, J. , Byrne, M. , Cunningham, E. , Amin, J. , Vickerman, P. , Martin, N. K. , Treloar, C. , Martinello, M. , Matthews, G. V. , Hajarizadeh, B. , & Surveillance and Treatment of Prisoners With Hepatitis C (SToP‐C) Study Group . (2022). Hepatitis C virus reinfection following direct‐acting antiviral treatment in the prison setting: The SToP‐C study. Clinical Infectious Diseases: An Official Publication of the Infectious Diseases Society of America, 75(10), 1809–1819. 35362522 10.1093/cid/ciac246

[bjhp70005-bib-0008] Caven, M. , Malaguti, A. , Robinson, E. , Fletcher, E. , & Dillon, J. F. (2019). Impact of hepatitis C treatment on behavioural change in relation to drug use in people who inject drugs: A systematic review. International Journal of Drug Policy, 72, 169–176. 31109776 10.1016/j.drugpo.2019.05.011

[bjhp70005-bib-0010] Dekkers, A. , Vos, S. , & Vanderplasschen, W. (2020). “Personal recovery depends on NA unity”: An exploratory study on recovery‐supportive elements in narcotics anonymous flanders. Substance Abuse Treatment, Prevention, and Policy, 15, 1–10. 32736568 10.1186/s13011-020-00296-0 PMC7393873

[bjhp70005-bib-0011] Dingle, G. A. , Cruwys, T. , & Frings, D. (2015). Social identities as pathways into and out of addiction. Frontiers in Psychology, 6, 1795. 26648882 10.3389/fpsyg.2015.01795 PMC4663247

[bjhp70005-bib-0012] Eddie, D. , Hoffman, L. , Vilsaint, C. , Abry, A. , Bergman, B. , Hoeppner, B. , Weinstein, C. , & Kelly, J. F. (2019). Lived experience in new models of Care for Substance use Disorder: A systematic review of peer recovery support services and recovery coaching. Frontiers in Psychology, 10, 1052. 31263434 10.3389/fpsyg.2019.01052 PMC6585590

[bjhp70005-bib-0013] Elbadry, M. , Badawi, M. , Youssef, N. , Duracinsky, M. , Saleh, S. S. , Funk, A. , Elessawy, H. , Rumpler, E. , Sayed, K. , Vasiliu, A. , Madec, Y. , Fontanet, A. , & El‐Kassas, M. (2024). Impact of treating chronic hepatitis C with direct acting antivirals on health‐related quality of life: A real‐life Egyptian experience. Egyptian Liver Journal, 14, 14.

[bjhp70005-bib-0014] Fraser, H. , Martin, N. K. , Brummer‐Korvenkontio, H. , Carrieri, P. , Dalgard, O. , Dillon, J. F. , Goldberg, D. , Hutchinson, S. , Jauffret‐Roustide, M. , Kåberg, M. , Matser, A. A. , Matičič, M. , Midgard, H. , Mravcik, V. , Øvrehus, A. , Prins, M. , Reimer, J. , Robaeys, G. , Schulte, B. , … Hickman, M. (2018). Model projections on the impact of HCV treatment in the prevention of HCV transmission among people who inject drugs in Europe. Journal of Hepatology, 68(3), 402–411. 29080808 10.1016/j.jhep.2017.10.010 PMC5841161

[bjhp70005-bib-0015] Goodyear, T. , Brown, H. , Browne, A. J. , Hoong, P. , Ti, L. , & Knight, R. (2021). ‘Stigma is where the harm comes from’: Exploring expectations and lived experiences of hepatitis C virus post‐treatment trajectories among people who inject drugs. International Journal of Drug Policy, 96, 103238. 33902968 10.1016/j.drugpo.2021.103238 PMC8881088

[bjhp70005-bib-0016] Goutzamanis, S. , Doyle, J. S. , Horyniak, D. , Higgs, P. , & Hellard, M. (2022). ‘Like a pickle that's been unpickled’: Emotional, identity and behavioural transformations throughout hepatitis C treatment. PLoS One, 17(12), e0272401. 36508406 10.1371/journal.pone.0272401 PMC9744280

[bjhp70005-bib-0017] Goutzamanis, S. , Horyniak, D. , Doyle, J. S. , Hellard, M. , Higgs, P. , & Treatment and Prevention study group . (2021). Perceived physical health outcomes of direct‐acting antiviral treatment for hepatitis C: A qualitative study. Harm Reduction Journal, 18, 73. 34266434 10.1186/s12954-021-00516-1 PMC8281623

[bjhp70005-bib-1003] Grebely, J. , Dalgard, O. , Conway, B. , Cunningham, E. B. , Bruggmann, P. , Hajarizadeh, B. , Amin, J. , Bruneau, J. , Hellard, M. , Litwin, A. H. , Marks, P. , Quiene, S. , Siriragavan, S. , Applegate, T. L. , Swan, T. , Byrne, J. , Lacalamita, M. , Dunlop, A. , Matthews, G. V. , … on behalf of the SIMPLIFY Study Group . (2018). Sofosbuvir and velpatasvir for hepatitis C virus infection in people with recent injection drug use (SIMPLIFY): An open‐label, single‐arm, phase 4, multicentre trial. The Lancet Gastroenterology & Hepatology, 3(3), 153–161. 29310928 10.1016/S2468-1253(17)30404-1

[bjhp70005-bib-0018] Guggisberg, H. , Nicca, D. , Kohler, A. , Bruggmann, P. , & Künzler‐Heule, P. (2022). “Shaping the new freedom”: A reflexive thematic analysis on patients' post cure needs after years of living with hepatitis C. Swiss Medical Weekly, 152(2324), w30177. 35704926 10.4414/smw.2022.w30177

[bjhp70005-bib-0019] Hagger, M. S. , & Orbell, S. (2021). The common‐sense model of illness self‐regulation: A conceptual review and proposed extended model. Health Psychology Review, 16(3), 347–377. 33461402 10.1080/17437199.2021.1878050

[bjhp70005-bib-0020] Hajarizadeh, B. , Cunningham, E. B. , Valerio, H. , Martinello, M. , Law, M. , Janjua, N. Z. , Midgard, H. , Dalgard, O. , Dillon, J. F. , Hickman, M. , Bruneau, J. , Dore, G. J. , & Grebely, J. (2020). Hepatitis C reinfection after successful antiviral treatment among people who inject drugs: A meta‐analysis. Journal of Hepatology, 72(4), 643–657. 31785345 10.1016/j.jhep.2019.11.012

[bjhp70005-bib-0021] Hamill, V. , Wong, S. , Kraiden, M. , Hayes, P. C. , Mutimer, D. , Yu, A. , Dillon, J. F. , Gelson, W. , Velásquez García, H. A. , Yeung, A. , Johnson, P. , Barclay, S. T. , Alvarez, M. , Toyoda, H. , Agarwal, K. , Fraser, A. , Bartlett, S. , Aldersley, M. , Bathgate, A. , … Innes, H. (2023). Mortality rates among patients successfully treated for hepatitis C in the era of interferon‐free antivirals population based cohort study. British Medical Journal, 382, e074001. 37532284 10.1136/bmj-2022-074001 PMC10394680

[bjhp70005-bib-0022] Harris, M. (2017). Managing expense and expectation in treatment revolution: Problematizing prioritization through an exploration of hepatitis C treatment ‘benefit’. International Journal of Drug Policy, 47, 161–168. 28455145 10.1016/j.drugpo.2017.03.015

[bjhp70005-bib-0023] Harris, M. , & Rhodes, T. (2018). “It's not much of a life”: The benefits and ethics of using life history methods with people who inject drugs in qualitative harm reduction research. Qualitative Health Research, 28(7), 1123–1134. 29557296 10.1177/1049732318764393

[bjhp70005-bib-0024] Hellard, M. , Schroeder, S. E. , Pedrana, A. , Doyle, J. , & Aitken, C. (2020). The elimination of hepatitis C as a public health threat. Cold Spring Harbor Perspectives in Medicine, 10(4), a036939. 10.1101/cshperspect.a036939 31712223 PMC7117951

[bjhp70005-bib-0025] Hickman, M. , Dillon, J. F. , Elliott, L. , De Angelis, D. , Vickerman, P. , Foster, G. , Donnan, P. , Eriksen, A. , Flowers, P. , Goldberg, D. , Hollingworth, W. , Ijaz, S. , Liddell, D. , Mandal, S. , Martin, N. , Beer, L. J. Z. , Drysdale, K. , Fraser, H. , Glass, R. , … Hutchinson, S. J. (2019). Evaluating the population impact of hepatitis C direct acting antiviral treatment as prevention for people who inject drugs (EPIToPe) – A natural experiment (protocol). BMJ Open, 9(9), e029538. 10.1136/bmjopen-2019-029538 PMC6773339 31551376

[bjhp70005-bib-0026] Krzeczkowska, A. , Flowers, P. , Chouliara, Z. , Hayes, P. , & Dickson, A. (2021). Experiences of diagnosis, stigma, culpability and disclosure in male patients with hepatitis C virus: An interpretative phenomenological analysis. Health, 25(1), 69–85. 31081379 10.1177/1363459319846939

[bjhp70005-bib-0027] Kwasnicka, D. , Dombrowski, S. U. , White, M. , & Sniehotta, F. (2016). Theoretical explanations for maintenance of behaviour change: A systematic review of behaviour theories. Health Psychology Review, 10(3), 277–296. 26854092 10.1080/17437199.2016.1151372 PMC4975085

[bjhp70005-bib-0028] Leventhal, H. , Phillips, L. A. , & Burns, E. (2016). The common‐sense model of self‐regulation (CSM): A dynamic framework for understanding illness self‐management. Journal of Behavioural Medicine, 39(6), 935–946. 10.1007/s10865-016-9782-2 27515801

[bjhp70005-bib-0029] Madden, A. , Hopwood, M. , Neale, J. , & Treloar, C. (2018). Beyond cure: Patient reported outcomes of hepatitis C treatment among people who inject drugs in Australia. Harm Reduction Journal, 15, 42. 30111327 10.1186/s12954-018-0248-4 PMC6094926

[bjhp70005-bib-0030] Martinello, M. , & Matthews, G. V. (2024). Reinfection after hepatitis C virus treatment—Keep testing, keep treating. JAMA Network Open, 7(8), e2430290. 10.1001/jamanetworkopen.2024.30290 39186277

[bjhp70005-bib-0031] McDonald, S. A. , Myring, G. , Palmateer, N. E. , McAuley, A. , Beer, L. , Dillon, J. F. , Hollingworth, W. , Gunson, R. , Hickman, M. , & Hutchinson, S. (2023). Improved health‐related quality of life after hepatitis C viraemic clearance among people who inject drugs may not be durable. Addiction, 118(7), 1340–1350. 36808787 10.1111/add.16169

[bjhp70005-bib-0032] National Harm Reduction Coalition . (2020). https://harmreduction.org/ (accessed 14 March 2025).

[bjhp70005-bib-0033] Palmateer, N. , Hamill, V. , Bergenstrom, A. , Bloomfield, H. , Gordon, L. , Stone, J. , Fraser, H. , Seyler, T. , Duan, Y. , Tran, R. , Trayner, K. , Biggam, C. , Smith, S. , Vickerman, P. , Hickman, M. , & Hutchinson, S. (2022). Interventions to prevent HIV and hepatitis C among people who inject drugs: Latest evidence of effectiveness from a systematic review (2011 to 2020). International Journal of Drug Policy, 109, 103872. 36202039 10.1016/j.drugpo.2022.103872

[bjhp70005-bib-0034] Pawlotsky, J. M. , Negro, F. , Aghemo, A. , Berenguer, M. , Dalgard, O. , Dusheiko, G. , Marra, F. , Puoti, M. , & Wedemeyer, H. (2020). European association for the study of the liver. EASL recommendations on treatment of hepatitis C: Final update of the series. Journal of Hepatology, 73(5), 1170–1218.32956768

[bjhp70005-bib-0035] Pourmarzi, D. , Smirnov, A. , Hall, L. , FitzGerald, G. , & Rahman, T. (2020). ‘I'm over the moon!’: Patient‐perceived outcomes of hepatitis C treatment. Australian Journal of Primary Health, 26(4), 319–324. 32580867 10.1071/PY20013

[bjhp70005-bib-0036] Prochaska, J. O. , & Velicer, W. F. (1997). The transtheoretical model of health behavior change. American Journal of Health Promotion, 12(1), 38–48. 10170434 10.4278/0890-1171-12.1.38

[bjhp70005-bib-0037] Pytell, J. D. , Sklar, M. D. , Carrese, J. , Rastegar, D. A. , Gunn, C. , & Chander, G. (2022). “I'm a survivor”: Perceptions of chronic disease and survivorship among individuals in long‐term remission from opioid use disorder. Journal of General Internal Medicine, 37(3), 593–600. 34027611 10.1007/s11606-021-06925-z PMC8141362

[bjhp70005-bib-0038] Richmond, J. A. , Ellard, J. , Wallace, J. , Thorpe, R. , Higgs, P. , Hellard, M. , & Thompson, A. (2018). Achieving a hepatitis C cure: A qualitative exploration of the experiences and meanings of achieving a hepatitis C cure using the direct acting antivirals in Australia. Hepatology, Medicine and Policy, 3, 8. 30288331 10.1186/s41124-018-0036-5 PMC6091021

[bjhp70005-bib-0039] Riemann, G. , & Schütze, F. (1991). Trajectory as a basic theoretical concept for analyzing suffering and disorderly social processes. In D. R. Maines (Ed.), Social organization and social process: Essays in honor of Anselm Strauss (pp. 333–357). de Gruyter.

[bjhp70005-bib-0040] Shiffman, M. L. (2018). The next wave of hepatitis C virus: The epidemic of intravenous drug use. Liver International, 38(Suppl. 1), 34–39. 29427493 10.1111/liv.13647

[bjhp70005-bib-0041] Van Bulck, L. , Luyckx, K. , Goossens, E. , Oris, L. , & Moons, P. (2019). Illness identity: Capturing the influence of illness on the person's sense of self. European Journal of Cardiovascular Nursing, 18(1), 4–6. 30392391 10.1177/1474515118811960

[bjhp70005-bib-0042] Vega, T. A. , Levander, X. A. , Seaman, A. , Korthuis, P. T. , & Englander, H. (2021). “Sobriety equals getting rid of hepatitis C”: A qualitative study exploring the interplay of substance use disorder and hepatitis C among hospitalized adults. Journal of Substance Abuse Treatment, 127, 108337. 34134860 10.1016/j.jsat.2021.108337 PMC8217723

[bjhp70005-bib-0043] Verma, D. , Ashkar, C. , & Saab, S. (2021). Cost effectiveness of direct acting antivirals in the treatment of hepatitis C in vulnerable populations. Expert Review of Pharmacoeconomics & Outcomes Research, 21(1), 9–12. 33073620 10.1080/14737167.2021.1838898

[bjhp70005-bib-0044] Whiteley, D. , Whittaker, A. , Elliott, L. , & Cunningham‐Burley, S. (2018). Hepatitis C in a new therapeutic era: Recontextualising the lived experience. Journal of Clinical Nursing, 27(13–14), 2729–2739. 10.1111/jocn.14083 28960567

[bjhp70005-bib-0045] World Health Organization . (2016). Global health sector strategy on viral hepatitis 2016–2021: towards ending viral hepatitis. WHO.

[bjhp70005-bib-0046] Yang, C. , Zhou, Y. , Cao, Q. , Xia, M. , & An, J. (2019). The relationship between self‐control and self‐efficacy among patients with substance use disorders: Resilience and self‐esteem as mediators. Frontiers in Psychiatry, 10, 388. 31249535 10.3389/fpsyt.2019.00388 PMC6582546

[bjhp70005-bib-0047] Yeung, A. , Palmateer, N. E. , Dillon, J. F. , McDonald, S. A. , Smith, S. , Barclay, S. , Hayes, P. C. , Gunson, R. N. , Templeton, K. , Goldberg, D. J. , Hickman, M. , & Hutchinson, S. J. (2022). Population‐level estimates of hepatitis C reinfection post scale‐up of direct‐acting antivirals among people who inject drugs. Journal of Hepatology, 76(3), 549–557. 34634387 10.1016/j.jhep.2021.09.038 PMC8852744

