# Supplementary material for: Post‐treatment life‐trajectories among people who inject drugs who completed hepatitis C treatment with direct acting antivirals: A thematic analysis
Source: Br J Health Psychol. 2025 Jul 11;30(3):e70005. doi: 10.1111/bjhp.70005 (PMC12254531; doi:10.1111/bjhp.70005)
Supplement: Supplementary file 1 — Data S1. [file BJHP-30-0-s001.docx]

**Topic Guide**

| **Introduction**  [explain purpose of research project and interview; check if participant feels comfortable; check regarding understanding confidentiality and the right to stop and/or withdraw from the study/interview, check regarding audio-recording; obtain informed consent] | |
| --- | --- |
| **1.** | **Tell me a little bit about what’s happened in your life since you completed your Hep C treatment?**   - Can you tell me a little about your drug use? - Who’s around for support at the moment? - What services do you use? (Probe for OST, mental health) - Why are you in touch with these services? |
| **2.** | **Can you sum up your Hep C treatment experience for me?**   - What was your end of treatment result? - What would you say to someone else who was thinking of having Hep C treatment?   - In a pharmacy?   - In a needle and syringe programme?   - In prison?   - In a drug treatment centre or clinic? |
| **3.** | **Since you’ve had the treatment, how has Hep C affected you?**   - What difference has it made to your life (if any)? - Did you have any expectations of the treatment? Have these been met? (Probe for both, negative and positive experiences) - Are there any downsides to being treated? (Probe for any changes including drug use/ injecting/ mental health) |
| **4.** | **Do any of your friends or family know about you having received Hep C treatment?**   - If so, tell me how it has affected them? - What difference has it made to their lives (if any)? |
| **5.** | **Have you been at risk of reinfection since you’ve had the Hep C treatment?**   - Can you tell me a bit more about the risks/ how you have avoided risks? - What do you do to keep safe? - What would you tell others if they want to keep themselves safe? [Probe for issues around harm reduction and Hep C testing and treatment] |
| **6.** | **If you had any top three tips in relation to getting tested and treated for Hep C what would they be?**   - What would you want to see improved? - How could Hep-C testing be better? - How could hep-C treatment be better? |
| **7.** | **If you had to think of the three things in Hep C testing and treatment to change, or to avoid, what would they be?**   - What would you want to see improved? - How could Hep-C testing be better? - How could hep-C treatment be better? |
| **8.** | [If the participant mentioned having had interferon Hep C treatment]  **How does your experience of the (new) Hep C treatment compare with the interferon treatment?**   - What was it like for you to be on Interferon? - How was that different from the new Hep C meds? - What is better? Why is that? - What is worse? Why is that? |
| **9.** | **Thinking back to your treatment, and your life in the last 12 to 24 months, is there anything else you want to say that we haven’t talked about?** |
| **End of interview**  [wrap up, thanking the participant; check participant wellbeing; end audio recording; explain and/or arrange access to vouchers] | |
